# Risperidone Effects on Brain Dynamic Connectivity—A Prospective Resting-State fMRI Study in Schizophrenia

**DOI:** 10.3389/fpsyt.2017.00014

**Published:** 2017-02-06

**Authors:** Kristin K. Lottman, Nina V. Kraguljac, David M. White, Charity J. Morgan, Vince D. Calhoun, Allison Butt, Adrienne C. Lahti

**Affiliations:** ^1^Department of Biomedical Engineering, University of Alabama at Birmingham, Birmingham, AL, USA; ^2^Department of Psychiatry and Behavioral Neurobiology, University of Alabama at Birmingham, Birmingham, AL, USA; ^3^Department of Biostatistics, University of Alabama at Birmingham, Birmingham, AL, USA; ^4^The Mind Research Network, Albuquerque, NM, USA; ^5^Department of Electrical and Computer Engineering, University of New Mexico, Albuquerque, NM, USA

**Keywords:** functional connectivity, dynamics, resting state, schizophrenia, antipsychotic medication

## Abstract

Resting-state functional connectivity studies in schizophrenia evaluating average connectivity over the entire experiment have reported aberrant network integration, but findings are variable. Examining time-varying (dynamic) functional connectivity may help explain some inconsistencies. We assessed dynamic network connectivity using resting-state functional MRI in patients with schizophrenia, while unmedicated (*n* = 34), after 1 week (*n* = 29) and 6 weeks of treatment with risperidone (*n* = 24), as well as matched controls at baseline (*n* = 35) and after 6 weeks (*n* = 19). After identifying 41 independent components (ICs) comprising resting-state networks, sliding window analysis was performed on IC timecourses using an optimal window size validated with linear support vector machines. Windowed correlation matrices were then clustered into three discrete connectivity states (a relatively sparsely connected state, a relatively abundantly connected state, and an intermediately connected state). In unmedicated patients, static connectivity was increased between five pairs of ICs and decreased between two pairs of ICs when compared to controls, dynamic connectivity showed increased connectivity between the thalamus and somatomotor network in one of the three states. State statistics indicated that, in comparison to controls, unmedicated patients had shorter mean dwell times and fraction of time spent in the sparsely connected state, and longer dwell times and fraction of time spent in the intermediately connected state. Risperidone appeared to normalize mean dwell times after 6 weeks, but not fraction of time. Results suggest that static connectivity abnormalities in schizophrenia may partly be related to altered brain network temporal dynamics rather than consistent dysconnectivity within and between functional networks and demonstrate the importance of implementing complementary data analysis techniques.

## Introduction

Schizophrenia is often described as a disorder of brain connectivity characterized by abnormal structural and functional network integration between cortical areas and likely related to clinical symptoms (1–3). A common approach to examining functional networks is through resting-state functional connectivity—the measure of temporal coherence of low frequency blood oxygenation level dependent (BOLD) signal fluctuations between spatially separate regions of the brain in the absence of a task being performed (1, 4, 5).

Traditionally, functional connectivity is evaluated over the length of the scan. In schizophrenia, a number of intrinsic network connectivity aberrations have been reported, albeit with widespread inconsistencies among studies (5–12). Importantly, this “static” approach to connectivity analysis disregards the dynamic nature of brain activity by assuming constant connectivity patterns over time (12, 13). Recent reports attribute inconsistencies across studies to the oversimplification of data in static functional connectivity analyses (12, 14–18). The recent emergence of dynamic functional connectivity analysis aims to address this data averaging issue by calculating transient patterns of functional connectivity through windowed time course sampling (12, 17, 19–24). Clustering of these patterns results in connectivity states that are believed to be representative of discrete mental states of connectivity that subjects pass through during the scan (12, 13, 25, 26). In capturing the fluctuations in network interactions over time, and ultimately more descriptively characterizing network integration, the progress toward identifying imaging biomarkers may be enhanced. However, the obstacle of determining the correct window size for sliding window analysis still remains. While some studies have indicated a window size between 30 and 60 s robustly estimates functional connectivity (24, 27), others have explored window sizes ranging from 30 to 240 s (12, 17, 19, 21, 23, 24, 28).

Prior resting-state functional connectivity studies reporting dysconnectivity in schizophrenia have been overwhelmingly obtained in medicated subjects. Because antipsychotic medications are known to affect brain activation (29–32), it is unclear to what extent prior findings are related to antipsychotic medication effects. In addition to examination of unmedicated patients with schizophrenia, functional connectivity studies of first episode schizophrenia patients and subjects at ultra-high risk of psychosis (33–35) allow for not only examination of earlier stages of psychosis but also non-confounded (i.e., antipsychotic medication exposure) functional connectivity abnormalities. More specifically Anticevic et al. and Cannon et al. (33, 35), found that subjects at ultra-high risk who converted to psychosis more prominently exhibited thalamocortical dysconnectivity, whereas Yoon and colleagues found that first episode and ultra-high risk subjects demonstrated frontotemporal dysconnectivity (34).

The purpose of this study was to evaluate dynamic functional network connectivity (dFNC) in unmedicated patients with schizophrenia and to test if, and how, antipsychotic medications change brain network dynamics after 1 and 6 weeks of treatment in an effort to disentangle medication effects form intrinsic illness characteristics. The corresponding description of dynamic connectivity patterns that may remain unaffected by antipsychotics could in turn contribute to the discovery of biomarkers for the development of new therapeutic interventions targeted at the other symptom domains (i.e., negative and cognitive symptoms) that are not alleviated by traditional antipsychotics (36). We hypothesized that static and dynamic analyses would provide complementary connectivity results. In accordance with previous studies examining temporal dynamics in schizophrenia (17), we also hypothesized that connectivity abnormalities would not be observed in all connectivity states; in addition, based on previous studies, we hypothesized that connectivity state abnormalities exhibited would be observed between thalamus and sensory network [i.e., auditory (AUD), visual (VIS), and somatomotor (SM)] connections (37, 38).

## Materials and Methods

### Participants

Thirty-four unmedicated patients with schizophrenia seeking treatment at the University of Alabama at Birmingham (UAB) were recruited from the emergency room, inpatient units, and various outpatient clinics. Additionally, 35 healthy controls matched on age, gender, smoking status, and socioeconomic status (SES) were recruited using flyers and advertisements in the university newspaper. This study was approved by the UAB Institutional Review Board and written informed consent for participation was obtained after participants were found competent to provide informed consent (39).

Diagnoses were established with review of medical records and evaluation by two board certified psychiatrists (Nina V. Kraguljac and Adrienne C. Lahti) and confirmed using the Diagnostic Interview for Genetic Studies (40). Patients included in the study had been off antipsychotic medication for at least 10 days; medication was not stopped to meet this criterion. Exclusion criteria were major medical conditions, neurological disorders, history of head trauma with loss of consciousness, substance abuse within 6 months of imaging (with the exception of nicotine), use of medication affecting brain function, pregnancy, and MRI contraindications. Healthy control exclusion criteria also included a history of Axis I disorders personally or in first-degree relatives.

Subjects who were either medication naïve or had been off antipsychotic medications were enrolled in a 6-week trial with risperidone using a flexible dosing regimen. Dose determinations were based on therapeutic and side effects. Starting doses were 1 mg; titration was done in 1–2 mg increments. Compliance was monitored by pill counts at each visit. Concomitant antidepressant or mood stabilizing medication was allowed to be used as indicated.

### Study Design

Participants completed a resting-state fMRI scan of at least 5 min (150 volumes) in length. For data length consistency across participants, additional volumes over 5 min were discarded (24). All participants were scanned at baseline. Patients were then scanned after 1 and 6 weeks of treatment to allow adequate time for clinical response (41, 42). Dropout and scanner intolerability are potential issues when working with a patient population. Therefore, of the 34 patients with schizophrenia enrolled, three subjects dropped out of the study prior to the week 1 scan and three more subjects dropped prior to the week 6 scan. One subject was excluded from baseline analysis due to an insufficient number of scan volumes. In addition, no resting-state scans were obtained for two subjects at week 1 and four subjects at week 6, leaving imaging data for 33 patients at baseline, 29 patients at week 1, and 24 patients at week 6 in the final analysis. Additionally, 19 of the 35 recruited controls were scanned for a second time 6 weeks after the baseline scan. Resting-state data from some subjects have been included in earlier reports (42–44). The Brief Psychiatric Rating Scale (BPRS) (45) was used to assess symptom severity weekly due to its briefer time to administer as time is critical when collecting data from unmedicated patients with schizophrenia. Cognitive function was assessed for both groups at baseline using the Repeatable Battery for the Assessment of Neuropsychological Status (RBANS) (46).

### Scanning Parameters

All scans were performed with a 3 T head-only scanner (Magnetom Allegra, Siemens Medical Solutions, Erlangen, Germany), with a circularly polarized transmit/receive head coil. High-resolution structural scans were acquired for anatomical reference using the 3-dimensional T1-weighted magnetization-prepared rapid acquisition gradient-echo sequence [repetition time/echo time/inversion time (TR/TE/TI) = 2300/3.93/1100 ms, flip angle = 12°, 256 × 256 matrix, 1-mm isotropic voxels]. Resting-state fMRI scans were acquired using a gradient recalled echo-planar imaging sequence (TR/TE = 2000/30 ms, flip angle = 70°, field of view = 192 mm × 192 mm, 64 × 64 matrix, 6 mm slice thickness, 1 mm gap, 30 axial slices). Participants were instructed to keep eyes open and stare passively ahead during the scan.

### Preprocessing

Data preprocessing was performed with SPM8 (Wellcome Trust Centre for Neuroimaging, London, UK^1^). Resting-state fMRI data were slice-timing corrected, realigned, normalized to Montreal Neurological Institute (MNI) space (47), resampled to 1.5 mm^3^, and smoothed with a Gaussian kernel to 6-mm full width at half maximum. Prior to group independent component analysis (ICA), data were variance normalized to facilitate decomposition of subcortical (SC) and cortical networks (17).

### Group ICA

Group-level spatial ICA was performed *via* the Group ICA of fMRI Toolbox (GIFT^2^) (48). Subject-specific principal component analysis (PCA) was implemented in the GIFT toolbox by reducing the data to 120 principal components, which were subsequently decomposed into 100 components *via* group data reduction (24). The expectation maximization algorithm was used to carry out PCA in a memory-efficient manner (24, 49). The Infomax algorithm (24, 50) was then applied to the PCA reduced data to generate 100 spatially independent components (ICs). Component stability/quality was measured by repeating the Infomax algorithm 20 times in ICASSO and the most representative run was used in subsequent steps (17, 24, 51). Subject-specific spatial maps and time courses were generated *via* GICA back-reconstruction (52). Scanning data for patients after 1 week of medication (*n* = 29) was also included in the group ICA and clustering analysis. Following back-reconstruction, subject spatial maps and time courses were scaled to *z*-scores.

### Postprocessing

**Figure 1:**
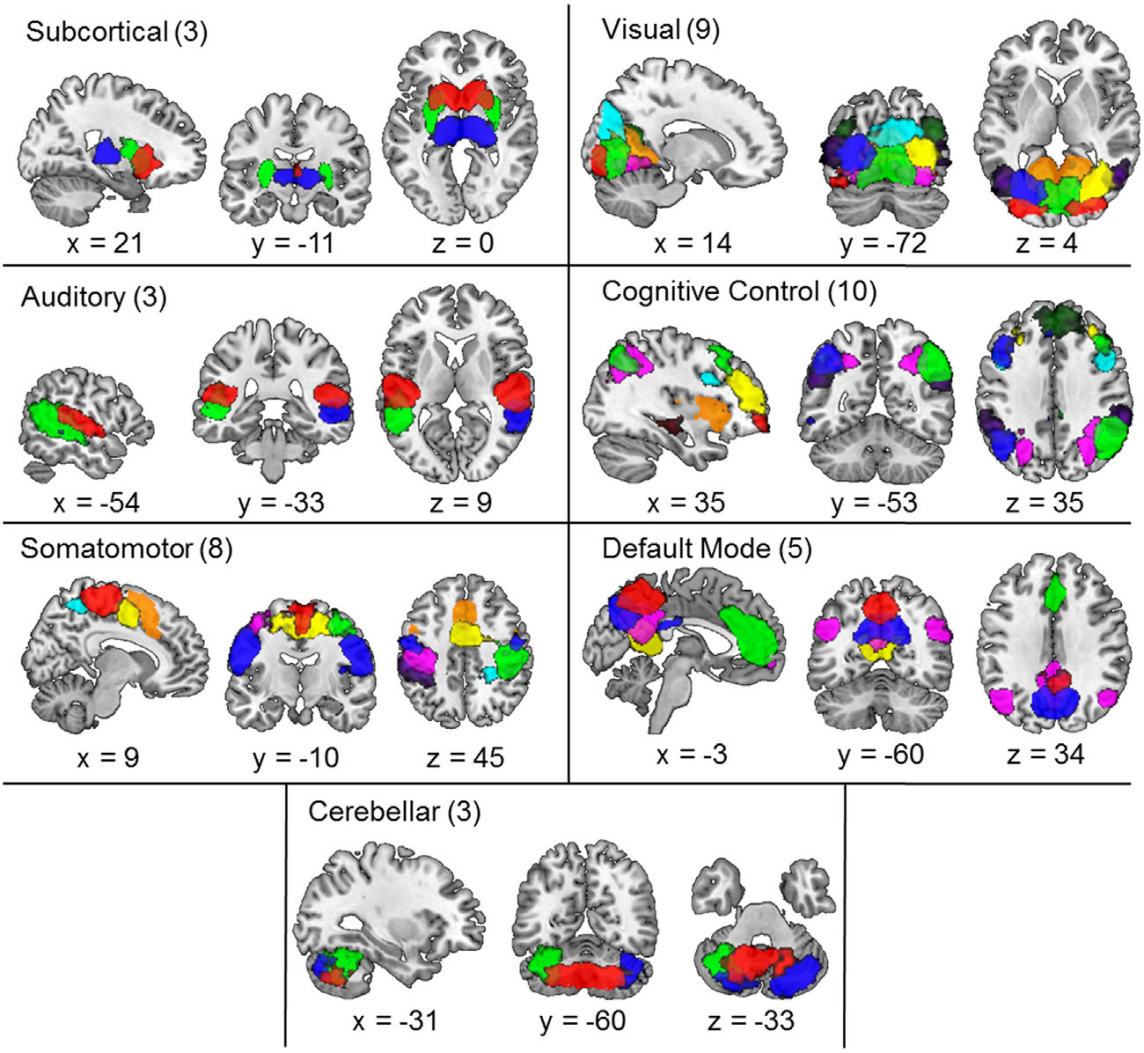
**Composite maps of the 41 independent components comprising resting-state networks extracted from the data *via* group independent component analysis and categorized into subcortical, auditory, somatomotor, visual, cognitive control, default mode, and cerebellar networks**. Each color in the composite map represents a different component and the number of components grouped in each category is indicated next to the category name. Peak activations of individual components can be seen in Table S1 in Supplementary Material.

Similar to procedures carried out in previous studies (17, 24, 53), three reviewers (Kristin K. Lottman, Nina V. Kraguljac, David M. White) collectively classified ICs as resting-state networks (RSNs)—as opposed to artifact—based on criteria-dependent visual inspection of group-level component spatial maps and evaluation of power spectra data. Group-level component spatial maps were inspected and classified as RSNs based on the criteria that peak activation clusters should occur primarily in gray matter, correspond anatomically to brain networks, and meet additional RSN expectations presented in (17, 24, 53). To facilitate component classification as RSNs, component power spectra data were evaluated using the fractional amplitude of low-frequency fluctuations (fALFFs) (53, 54) in order to validate that component time courses were characterized by predominantly low-frequency fluctuations (24, 55, 56). Based on reviewer consensus, the three reviewers collectively identified 41 group-level RSNs from the 100 extracted components, as illustrated in Figure 1. RSN labels were identified based on results from brain atlas toolboxes utilized in SPM8—xjView^3^ and WFU_PickAtlas^4^. Additionally, RSN labels were determined based on correspondence to the 50 components presented in (24), as well as label consensus among all three reviewers (see Table S1 in Supplementary Material for RSN peak activations). Labeled RSNs were then organized into seven different networks including SC, AUD, SM, VIS, cognitive control (CC), default mode (DM), and cerebellar (CB) (24).

Following RSN identification, framewise displacement was regressed from the subject-specific RSN time courses prior to static and dynamic connectivity analyses. Framewise displacement was computed as the absolute frame-to-frame displacement of the brain from the six realignment parameters using a radius of 50 mm to convert angle rotations to displacements (42, 57). Ultimately, this resulted in individual displacement values for each volume of the time course (i.e., 150 frame-to-frame displacement values—with an initial displacement value of 0—were used as regression covariates for each subject). While controls and unmedicated patients (baseline) exhibited differences in mean framewise displacement (*F* = 6.867, *p* = 0.011) as expected, patients did not demonstrate significant differences in mean framewise displacement from baseline to week 6 (*F* = 0.200, *p* = 0.659) or week 1 to week 6 (*F* = 3.517, *p* = 0.075). However, mean framewise displacement was found to be significantly different between baseline and week 1 patients (*F* = 4.325, *p* = 0.047) Subsequently, subject-specific RSN time courses were detrended, despiked, and band-pass filtered (0.01–0.15 Hz) using a fifth order Butterworth filter in accordance with previous studies (17, 24).

### Static Functional Network Connectivity Analysis

**Figure 2:**
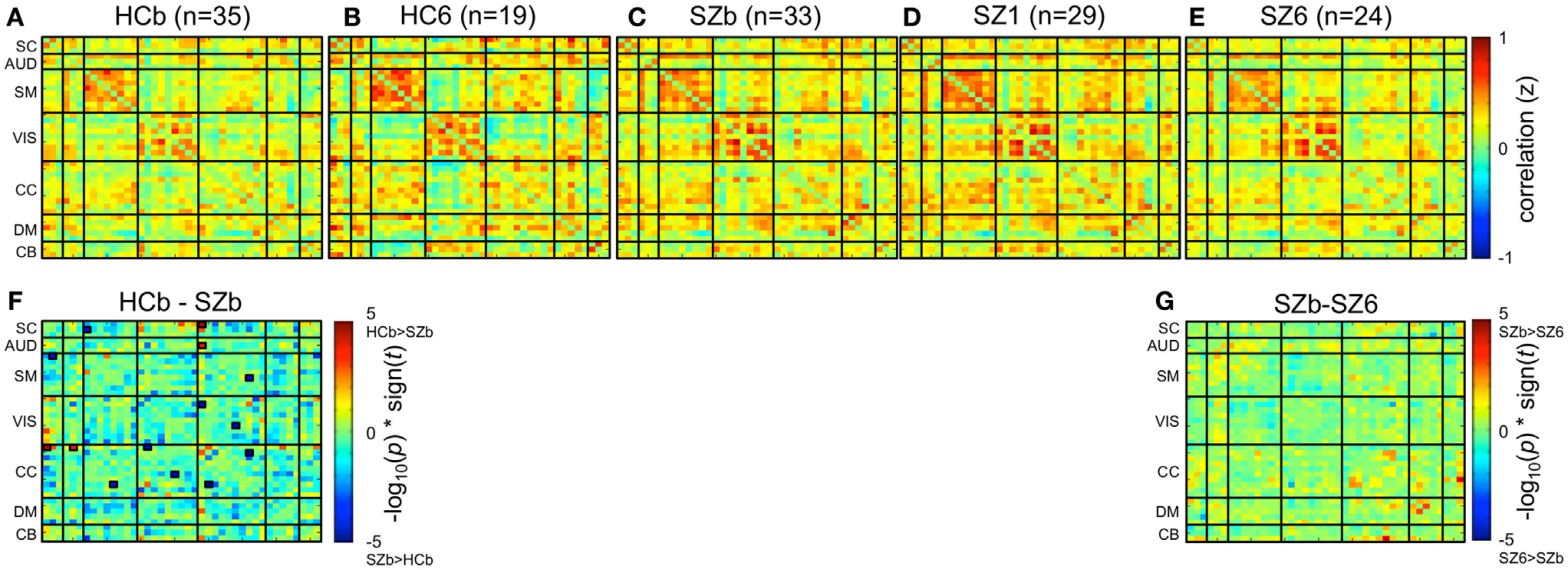
**Static functional network connectivity**. Group-level mean static functional network connectivity for **(A)** 35 controls at baseline (HCb), **(B)** 19 controls after 6 weeks (HC6), **(C)** 33 unmedicated patients with schizophrenia (SZb), **(D)** 29 patients after 1 week of medication (SZ1), and **(E)** 24 patients after 6 weeks of medication (SZ6). Group differences are shown between **(F)** controls and unmedicated patients (HCb-SZb) and **(G)** unmedicated patients and week 6 patients (SZb-SZ6). Significant group differences outlined with a small black box are indicated if *p*_FDR_ < 0.05. No significant differences were observed when week 1 patients were compared to baseline and week 6 patients.

Static functional network connectivity was estimated for each subject as the pairwise Pearson correlation between whole RSN component time courses, resulting in a 41-by-41-component *z*-scored correlation matrix for each subject. Correlation matrices for subjects in a group were then averaged together resulting in a group-level connectivity matrix (Figures 2A–E). Subsequently, within and between-group differences in static functional network connectivity matrices were evaluated on subject-level matrices *via* respective paired and two-sample univariate *t*-tests with a significance value of *p_*False Discovery Rate (FDR)*_* < 0.05 (Figures 2F,G).

### dFNC Analysis

**Figure 3:**
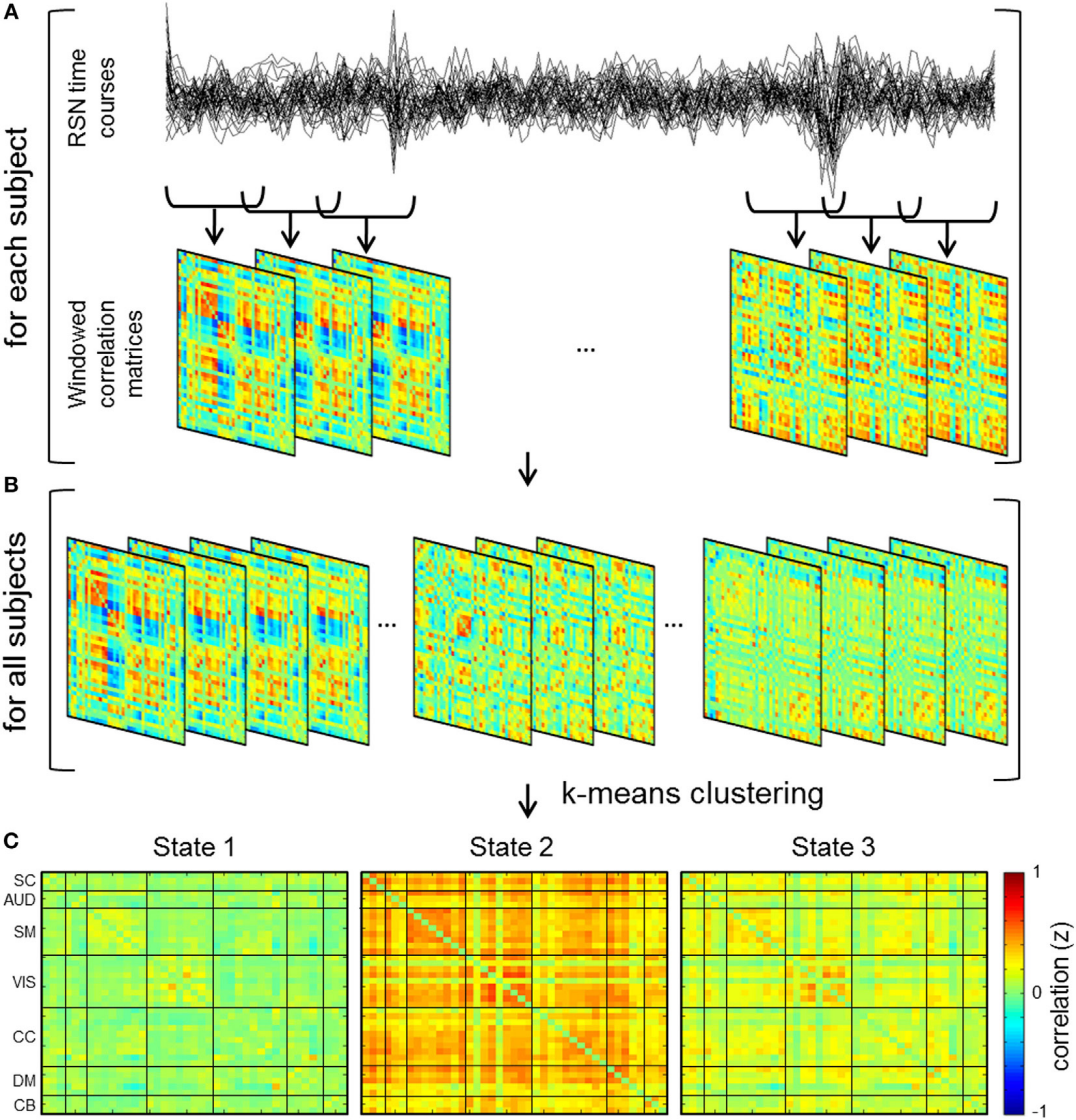
**Schematic depicting dynamic functional network connectivity (dFNC) analyses**. **(A)** dFNC analysis computes functional network connectivity on windows of the independent component time courses and hence windowed correlation matrices are generated for each subject. **(B)** Concatenation of dFNC windows for all subjects and subsequent *k*-means clustering of the windows results in cluster centroids or connectivity states. **(C)** dFNC cluster centroids for 44 s window.

A sliding window technique was used to estimate dFNC where windowed segments of the component time courses were used to compute transient functional network connectivity patterns (Figure 3). Sliding window analysis was iteratively performed with window sizes of 30, 40, 44, 50, and 60 s. Window sizes were selected based on previous studies indicating functional connectivity can be robustly estimated using a window size between 30 and 60 s (24, 27), as well as previous implementation of a 44 s window in a dataset of patients with schizophrenia (17). A Gaussian (σ = 3 TRs) window of the respective size was slid through the time course in steps of one TR in order to obtain windowed correlation matrices for each subject (Figure 3A). Due to potential effects of noise on covariance estimation from sampling short time windows, windowed correlation matrices were generated by estimating the covariance of the L1 regularized inverse covariance matrix, which was carried out utilizing a graphical LASSO framework in the GIFT Toolbox (17, 24, 58–60).

### Window Size Classification

A 44 s window was chosen based on implementation in previous studies in a similar subset of subjects. Subsequently, similar to a previous study (61), the optimal window size of 44 s for dynamic analyses was validated by the accuracy of a linear support vector machine (SVM) classifier in predicting group (i.e., controls and unmedicated patients) based on the amplitude of low-frequency fluctuation of functional connectivity (ALFF-FC) for each subject and respective window sizes. ALFF-FC is utilized in order to demonstrate the variability in network connections over time (61). In order to calculate the ALFF-FC, the fast fourier transform (FFT) was applied to the windowed correlation values for each subject (61). Subsequently, the FFT values divided by the largest frequency value (i.e., 0.5 Hz) that fell within the frequency band of 0.01–0.08 Hz were summed to obtain ALFF-FC maps for each subject (61).

A linear SVM classifier was utilized to discriminate between controls and unmedicated patients. A leave-one-out cross validation (LOOCV) method was applied to determine the average accuracy values of the SVM classifier. For each iteration of LOOCV, one sample is designated as a test sample and the remaining samples are designated as the training set. A two-sample t-test between the ALFF-FC values of controls and unmedicated patients in the training set was performed at each LOOCV fold (61). In order to reduce the number of features, as well as the possibility of overfitting, only the significant resultant values (*p* < 0.05) were selected as predictor features (61). Therefore, the number of predictor features varied from fold-to-fold of the LOOCV. Additionally, SVM classification was only performed to discriminate between controls and unmedicated patients; thus, only 68-folds of the LOOCV were carried out. SVM classifier analysis was performed on dynamic data from implementation of the five different window sizes (30, 40, 44, 50, and 60 s). The optimal window size was determined as the window size with the best accuracy in differentiating control and patient ALFF-FC using the SVM classifier. This SVM classification was performed using the Statistics and Machine Learning Toolbox in MATLAB (62).

### Clustering

In order to characterize reoccurring patterns of connectivity across groups and time, *k*-means clustering was performed on the windowed correlation matrices for all subjects (Figure 3B). Clustering of a sub-sampled number of windows (i.e., windows with relative maxima of variance) for all groups and time points was carried out in order to estimate initial cluster centroids (cluster medians) (17, 24). The sum of absolute differences or L1 distance method was used with a maximum of 150 iterations for *k*-means cluster computation. The optimal number of cluster states was determined to be three based on evaluation of the elbow criterion of the ratio of within cluster sum of squares distance to between cluster sum of squares distance (17, 63). More specifically, the number of clusters is determined as the point or “elbow” in the plot (i.e., ratio by cluster number) followed by a flattening of the plot where increasing number of *k* clusters provides marginal information (64). Resultant centroid states from the clustering of sub-sampled data were subsequently used as initial clustering positions for clustering of all subject and group data.

### Group Differences in dFNC

Following *k*-means clustering of data from all subjects mean group-level connectivity centroid states were calculated from the group’s subject medians of windows assigned to each respective state (17). Subsequently, respective paired and two-sample univariate *t*-tests were performed on the subject-level connectivity states to evaluate group differences. Group differences of *p_FDR_* < 0.05 were considered significant. It is important to note that implementation of L1 regularization using the graphical LASSO framework for dynamic windowing resulted in small variations in windowed correlations from run to run. Therefore, in order to account for potential fluctuations in significant group differences from iteration to iteration, as well as determine connectivity reliability, bootstrap resampling (i.e., resampling with replacement) was conducted on clustered windowed correlation values with a resampling rate of 10,000. Group differences (*p_FDR_* < 0.05) that occurred in at least 95% of the 10,000 bootstrap resamples were considered significant.

Exploratory *post hoc* analyses examining group differences in state statistics including dwell times (i.e., average amount of time spent occupying a state before switching to another) and overall amount of time spent in a state were implemented using two-sample and paired univariate *t*-tests, where appropriate. Transition matrix differences for each group were also evaluated *via* chi-square methods (65–67). More specifically, transition matrices represent the probability of transitioning from one state to another (e.g., state 1 to state 2, etc.) (24). Additionally, the relationship between clinical improvement and state statistics were examined *via* correlation analysis of mean dwell time/fraction of time spent in a state and treatment response. Treatment response was defined as the percent change in positive BPRS scores from baseline to week 6. In order to demonstrate the complementary nature of connectivity analyses, the effectiveness of static, dynamic, and both static and dynamic connectivity analyses in classifying controls and patients was evaluated *via* group classification using a linear SVM and LOOCV. Significant connectivity values, fraction of time spent in a state, and mean dwell time were used as predictor variables. More specifically, the effectiveness of static connectivity analyses was evaluated with seven connectivity predictor variables. The effectiveness of dynamic connectivity analyses was evaluated with fraction of time spent in a state and mean dwell time values (i.e., six predictor variables). Both static and dynamic connectivity analysis accuracy was evaluated with all aforementioned predictor variables (i.e., 13 predictor variables).

## Results

### Demographics

No significant differences in age, gender, parental SES, smoking status, or daily cigarette use were observed between controls and patients. Patients exhibited a decrease in total BPRS scores from 48.29 ± 9.38 at baseline to 30.57 ± 8.47 after 6 weeks of medication. Average dose of risperidone was 4.36 ± 1.45 mg at the week 6 scan. Twelve subjects were concomitantly treated with benztropine, two with trazodone, one each was prescribed mirtazapine, amitriptyline, and valproic acid. In comparison to healthy controls, patients scored significantly lower on RBANS (Table 1).

**Table 1:** **Demographics and clinical assessments^a^**.

	HC (*n* = 35)	SZ (*n* = 34)	*t*/χ^2^	*p*-Value
**Age (years)**	32.00 ± 8.90	32.38 ± 10.43	−0.164	0.87
Gender (male/female)	25/10	23/11	0.116	0.733
Parental SES^b^	5.80 ± 4.21	7.26 ± 6.39	23.17	0.058
Smoking status (Y/N)	22/13	26/8	1.51	0.219
Smoking (packs per day)	0.61 ± 0.61	0.59 ± 0.53	0.168	0.867
**Diagnosis**				
Schizophrenia	–	31	
Schizoaffective disorder	–	3	
**Illness characteristics**				
Illness duration (years)	–	9.59 ± 9.94	
First episode	–	12	
**Prior antipsychotic treatment**				
Antipsychotic naïve	–	17	
Antipsychotic-free interval (months)	–	23.08 ± 44.42	
**Baseline BPRS^c^ (***n*** = 34)**				
Total score	–	48.29 ± 9.38		
Positive symptom subscale	–	9.53 ± 3.04		
Negative symptom subscale	–	6.79 ± 2.51		
**Week 6 BPRS (***n*** = 28)**				
Total score	–	30.57 ± 8.47		
Positive symptom subscale	–	4.86 ± 2.38		
Negative symptom subscale	–	5.39 ± 2.42		
**RBANS**				
Total index	93.74 ± 14.33	70.21 ± 13.76	6.96	<0.001
Immediate memory	95.74 ± 12.73	74.68 ± 16.86	5.87	<0.001
Visuospatial	87.26 ± 19.35	71.41 ± 15.48	3.75	<0.001
Language	100.2 ± 14.04	84.71 ± 12.85	4.78	<0.001
Attention	100.34 ± 19.33	79.03 ± 20.32	4.47	<0.001
Delayed memory	93.06 ± 11.83	72.53 ± 19.10	5.35	<0.001

*HC, healthy control; SZ, schizophrenia; SES, socioeconomic status; Y, yes; N, no; BPRS, Brief Psychiatric Rating Scale; RBANS, Repeated Battery for the Assessment of Neuropsychological Status*.

*^a^Mean ± SD unless otherwise indicated*.

*^b^SES ranks reported from Diagnostic Interview for Genetic Studies scale (1–18); high rank (lower numerical value) corresponds to high SES status. Data unavailable for seven participants (one HC, six SZ)*.

*^c^BPRS reported on 1–7 scale; positive (conceptual disorganization, hallucinatory behavior, and unusual thought content); negative (emotional withdrawal, motor retardation, and blunted affect)*.

### RSN Identification

The 41 maximally ICs identified as RSNs are depicted in Figure 1. Labeled RSNs were then organized into seven different networks including SC (3 RSNs), AUD (3 RSNs), VIS (9 RSNs), SM (8 RSNs), CC (10 RSNs), DM (5 RSNs), and CB (3 RSNs; Table S1 in Supplementary Material).

### Window Size Classification

The 44-s window size is in accordance with the window size implemented in previous studies examining dynamic connectivity in controls (24) and in patients with schizophrenia (17). The optimal window size was validated to be 44 s since classifier accuracy was highest (i.e., 77.94%) at this window size. A 10,000-iteration permutation test indicated the probability of obtaining this accuracy value with a 44 s window was not by chance (*p* < 0.05) (61).

### Group Differences in Static Functional Network Connectivity

#### Between Group Differences

Mean static functional network connectivity matrices for baseline controls, week 6 controls, unmedicated patients, week 1, and week 6 patients are illustrated in Figures 2A–E. In comparison to controls, unmedicated patients demonstrated increased connectivity within the CC network and between the SC-SM, VIS-CC, and SM-CC network connections, but decreased connectivity between the SC-CC and AUD-CC networks (Figure 2F).

#### Connectivity Changes over Time

No significant differences (*p*_FDR_ > 0.05) in connectivity were exhibited when comparing unmedicated and week 6 medicated patients (Figure 2G), as well as baseline and week 6 controls. Additionally, week 1 patients demonstrated no significant connectivity differences (*p*_FDR_ > 0.05) when compared to baseline and week 6 patients.

### Group Differences in dFNC

#### Between Group Differences

Cluster centroids for all subjects and time points are shown in Figure 3C. Three discrete connectivity states, a relatively sparsely connected state (State 1), a relatively abundantly connected state (State 2), and an intermediately connected state (State 3) were identified. No significant differences (*p*_FDR_ > 0.05) in connectivity were exhibited when comparing controls to unmedicated patients, week 1 patients, and week 6 patients. While bootstrap resampling of windowed correlations was implemented to determine connectivity stability/reliability, analyses resulted in no significant connectivity differences in more than 95% of the 10,000 resamples. It is important to note that, in comparison to controls, unmedicated patients exhibited some instances of hyperconnectivity (*p*_FDR_ < 0.05) between SC (i.e., IC 45 (thalamus) and SM network connections in the sparsely connected state (State 1) among the 10,000 resamples.

#### Connectivity Changes over Time

Evaluation of significant state connectivity differences across time in patients with schizophrenia indicates no significant change in connectivity (i.e., hyper- or hypo-connectivity—between baseline and week 1, baseline and week 6, and week 1 and week 6 over time (*p*_FDR_ < 0.05)). In addition, no significant connectivity differences (*p*_FDR_ < 0.05) were exhibited between baseline and week 6 in controls.

#### Connectivity State Statistics

**Figure 4:**
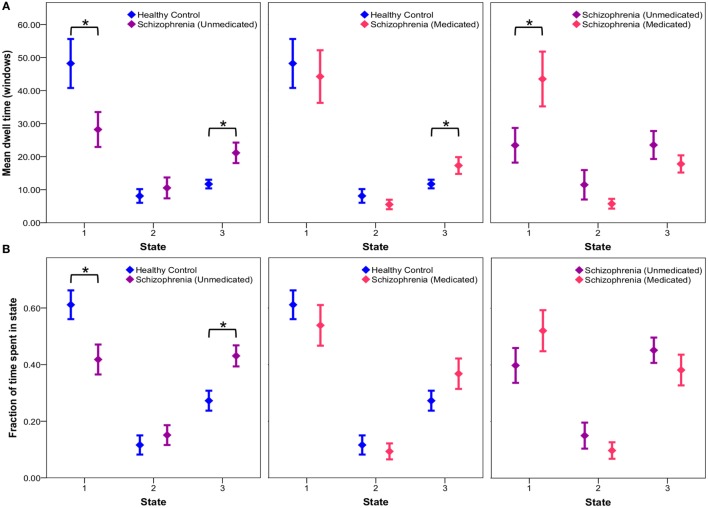
**Connectivity state statistics**. Exploratory *post hoc* analysis of mean dwell time **(A)** and fraction of time **(B)** subjects spend in each state at each window size. Mean group dwell times **(A)** and mean fraction of time groups spend in a state **(B)** are depicted with error bars representing the standard error of the mean. Significant group differences (*p* < 0.05) obtained *via* respective two-sample and paired *t*-tests are indicated with asterisks.

Exploratory *post hoc* analyses reveal no significant group differences in transition probabilities between states across groups. Comparison of mean dwell times indicates that unmedicated patients tend to dwell in the sparsely connected state 1 for significantly less time (*p* = 0.0334) and the intermediate state 3 for significantly more time (*p* = 0.0055) than controls (Figure 4). After 6 weeks of risperidone treatment, state 1 dwell times significantly increased in patients (*p* = 0.0115), but state 3 dwell times remain unchanged (*p* = 0.1718) in comparison to unmedicated patients (Figure 4). Additionally, comparison of the fraction of time groups occupy in individual states indicate that unmedicated patients occupy state 1 significantly less (*p* = 0.0105) and state 3 significantly more than controls (*p* = 0.0029), but this was not affected by treatment (*p* > 0.05). Comparison of healthy controls over time indicates no significant differences in mean dwell time (*p* > 0.05) and fraction of time spent in a state (*p* > 0.05) for all three states (intraclass correlation coefficients for controls over time presented in Table S2 in Supplementary Material). Patients after 1 week of medication demonstrated no significant differences in mean dwell time (*p* > 0.05) and fraction of time spent in a state (*p* > 0.05) for all three states when compared to patients at baseline and week 6. See Table S3 in Supplementary Material for all *post hoc* connectivity state statistics.

Additional exploratory *post hoc* analyses of clinical symptoms revealed no significant correlation between dwell times/fraction of time spent in a state and treatment response. Group classification using linear SVMs demonstrated a higher effectiveness (i.e., classification accuracy) of 83.8% (sensitivity: 91.4%; specificity: 75.8%) when incorporating both static and dynamic data as predictor variables, compared to the utilization of individual static (accuracy: 73.5%; sensitivity: 80%; specificity: 67%) and dynamic (accuracy: 58.8%; sensitivity: 74.3%; specificity: 42.4%) predictor variables.

## Discussion

To our knowledge, this is the first resting-state functional connectivity study examining brain network temporal dynamics in unmedicated patients with schizophrenia and the effects of antipsychotic medication. We describe widespread static connectivity abnormalities, both hyper- and hypo-connectivity, between controls and unmedicated patients with schizophrenia. Dynamic analyses suggest three discrete connectivity states, a relatively sparsely connected state, a relatively abundantly connected state, and an intermediate state. Significantly increased connectivity was present only between the thalamus and SM network in one state in unmedicated patients compared to controls, but we found no evidence of decreased connectivity in any states. Exploratory analyses of state statistics indicate that, in comparison to controls, unmedicated patients have shorter mean dwell times and fraction of time spent in the sparsely connected state, and longer dwell times and fraction of time spent in the intermediately connected state. Risperidone appears to normalize mean dwell times, but not fraction of time spent.

In unmedicated patients, our static functional network connectivity results reveal altered patterns of functional connectivity within the CC network and between the SM-SC, CC-VIS, AUD-CC, SM-CC, and CC-SC networks. These results are in line with recent studies in medicated patients demonstrating altered systems-level brain network dysfunction that suggest impaired integration within and between bottom-up and top-down networks (38, 68). Similar to studies that use a seed-based (37, 69) or static and dynamic (17) functional connectivity approaches in medicated patients, we observed increased SC-SM static connectivity in unmedicated patients compared to controls. Likewise, previous studies in subjects at ultra-high risk of psychosis demonstrated thalamocortical (33, 35) and frontotemporal (34) dysconnectivity. Interestingly, risperidone did not appear to change this SC-SM dysconnectivity pattern, despite several recent reports that suggest that antipsychotic medications may affect functional connectivity (31, 44, 70–72). However, it is important to note that although a similar sample was utilized in (44), disparate findings may be attributable to varying analysis techniques.

Although widespread dysconnectivity in schizophrenia is reported throughout the literature [see Pettersson-Yeo et al. (73), for review], a large portion of the literature reports decreased functional connectivity strength in patients with schizophrenia compared to controls, as well as the involvement of prefrontal brain region connections (3, 32, 73, 74). In comparison, our static connectivity results (with the exception of a CC-SC and AUD-CC connection) demonstrate increased connectivity in patients compared to controls. While differences in our dysconnectivity results may be attributable to the inclusion of an unmedicated population, as well as variable experimental design and analysis, the heterogeneity of the disorder and inconsistencies in the underlying neural mechanisms may also impact the functional outcome (73–76).

Dynamic connectivity analyses only replicate thalamus-SM hyperconnectivity found in static analyses in one of the three connectivity states, but do not demonstrate evidence of dysconnectivity within or between any other RSNs in any state. While the exact etiology of the connectivity states presented in this work is unknown, recent studies have reported that connectivity states may correspond to stages of consciousness (13, 26). Therefore, these characteristic state-dependent connectivity patterns exhibited in dynamic analysis are promising for future identification of potential imaging biomarkers representative of the disorder of schizophrenia (13). The limited evidence of connectivity abnormalities in dynamic analyses may very well be a more comprehensive illustration of connectivity abnormalities in comparison to analyses that “oversimplify” the data with static time course assumptions (12, 13).

Exploratory *post hoc* analyses of state statistics revealed that controls spend the majority of time in a sparsely connected state, while unmedicated patients with schizophrenia do so less. In accordance with these results, mean dwell time analysis demonstrated that controls tend to dwell for a significantly longer time in the sparsely connected state compared to unmedicated patients; however, patient dwell time in the sparsely connected state normalizes after 6 weeks of risperidone treatment. It is tempting to speculate that dwell time abnormalities may be related to disorganized patterns of neuronal activity, potentially secondary to glutamatergic hyperactivity (77) thought to be present in unmedicated patients with schizophrenia (78–80). Conceivably, this disorganized firing pattern could result in patients being unable to reside in a sparsely connected state for extended periods of time leading to an impaired ability to filter out irrelevant information. Alternatively, dopaminergic hyperactivity attenuated by antipsychotic medication may also explain findings. Pharmacological animal models of schizophrenia have demonstrated restoration of cortical synchronization with antipsychotic medications (81, 82), which could in turn result in dwell time normalization to control levels following risperidone treatment.

The lack of connectivity abnormalities before and after antipsychotic medication in the presence of medication-dependent dwell time abnormalities further substantiate the non-specific impact of antipysychotic medications on functional connectivity illustrated by Lui and colleagues (31). Additionally, these results are not discernible in static analyses, therefore, indicating the value of assessing functional connectivity dynamically in order to more accurately distinguish patient populations (13).

The differences in connectivity demonstrated in static analyses, as well as dwell time and fraction of time abnormalities in dynamic analyses, not only reiterate the advantage of the dynamic approach to examining functional connectivity but may also suggest the complementary nature of static and dynamic functional connectivity analyses (13, 83). Additionally, *post hoc* classification analyses support this complementary relationship. Based on this information, future studies would benefit from utilizing both static and dynamic analyses for assessing functional connectivity (13).

Several strengths and limitations have to be considered in the interpretation of our findings. To avoid the confounding effects of medication on functional connectivity, we only enrolled subjects free of exposure to antipsychotic medications for at least 10 days preceding the baseline scan. To minimize variance in the data, we carefully matched groups on several factors including parental SES and smoking, did rigorous preprocessing, and used a longitudinal design with a single antipsychotic medication to evaluate whether baseline dysconnectivity patterns normalized with treatment. In addition, we controlled for the effect of time on functional connectivity by scanning a group of matched controls 6 weeks apart. A sliding window analysis was implemented with a window size of 22 TRs (44 s) in order to estimate connectivity dynamics. Previous studies have indicated a window size between 30 and 60 s robustly estimates functional connectivity (24, 27). In addition, Telesford and colleagues demonstrated that smaller window sizes are more sensitive to detecting individual differences, whereas group-level differences can be better estimated at larger window sizes (84); however, a standard window size has yet to be established. While we are confident in our machine learning approach to validating the optimal window size, future work exploring potential data-driven window size determination methods would be welcomed. Similarly, time-frequency approaches may also be useful as such approaches do not require windowing (85). Furthermore, physiological artifacts such as heart rate and breathing were not directly controlled for during acquisition. Although image preprocessing and ICA indirectly controls for these, results may be impacted by physiological and motion artifacts. While the complementary nature of static and dynamic analyses of resting-state fMRI was demonstrated *via* utilization of a linear SVM (i.e., 83.8% classification accuracy), future studies utilizing multimodal data may further increase this classification accuracy (86). Although the sample size used in this study is sufficient for robustly estimating static functional connectivity, state connectivity patterns and group differences may be impacted from an inadequate number of subjects exhibiting certain states. Additionally, due to the complex nature of dynamic connectivity patterns, development of multifaceted statistics to capture these complexities would be advantageous. For example, current analyses restrict subjects to exhibiting a single connectivity state at a specific time when there may in fact be an overlap in connectivity state manifestation. The ability to capture potentially overlapping connectivity states (13, 87–90) may provide critical information to ultimately understanding the intricacies of brain function. It is also important to note that the choice of the frequency band utilized when filtering data may impact functional connectivity (91), as well as the classification accuracy in differentiating patients and controls (92). Due to the lack of a placebo group in this study, changes in functional connectivity cannot definitively be characterized as medication effects. In addition, schizophrenia is a highly heterogeneous disorder in which there are likely multiple pathological mechanisms causing patients to react differentially to antipsychotic medications. Therefore, until the underlying pathological mechanisms and ultimately the heterogeneity of the disorder are identified, analysis results may continue to remain variable as no one specific analysis technique can be distinguished as optimal.

Our results suggest that static connectivity abnormalities in schizophrenia may partly be related to altered brain network temporal dynamics rather than dysconnectivity of within and between functional networks alone. Medications appear to partially attenuate, but not fully reverse, brain network dynamic alterations, suggesting that dynamic connectivity could be leveraged as biomarker for the development of novel treatments targeted toward symptom dimensions that are unaffected by antipsychotic medications. Ultimately, our study highlights the importance of implementing complementary data analysis techniques; the additional information provided by dynamic analyses may be used in the advancement toward identification of imaging biomarkers.

## Clinical Trials Registration

Registry Name: Treatment Response in Schizophrenia: Bridging Imaging and Postmortem Studies. URL: https://clinicaltrials.gov/ct2/show/NCT00937716?term=NCT00937716&rank=1. Registration Number: NCT00937716.

## Ethics Statement

This study was carried out in accordance with the recommendations of the University of Alabama at Birmingham Institutional Review Board with written informed consent from all subjects. All subjects gave written informed consent in accordance with the Declaration of Helsinki. The protocol was approved by the University of Alabama at Birmingham Institutional Review Board.

## Author Contributions

AL designed the study. AL, NK, and AB recruited patients. NK and DW collected the fMRI data. KL performed analyses and wrote the manuscript with the help of NK, DW, and AL. CM aided in statistical analyses. VC provided technical advice for dynamic analysis. All authors contributed to and approved the final version of this manuscript.

## Conflict of Interest Statement

Medication was donated by Janssen Pharmaceuticals, Inc., Dr. AL has received funds from Janssen Pharmaceuticals, Inc., as part of an investigator-initiated study. Dr. AB, Dr. VC, Dr. NK, KL, Dr. CM, and DW declare no potential conflicts of interest.

## Footnotes

^1^http://www.fil.ion.ucl.ac.uk/spm/.

^2^http://mialab.mrn.org/software/gift.

^3^http://www.alivelearn.net/xjview8/.

^4^http://www.nitrc.org/projects/wfu_pickatlas/.
