# Supplementary material for: Risperidone Effects on Brain Dynamic Connectivity—A Prospective Resting-State fMRI Study in Schizophrenia
Source: Front Psychiatry. 2017 Feb 6;8:14. doi: 10.3389/fpsyt.2017.00014 (PMC5292583; doi:10.3389/fpsyt.2017.00014)
Supplement: Supplementary file 1 [file Table_1.DOCX]

Table S1: RSN Peak Activations

| RSN regions | T_max_ | Peak Coordinates^1^ | | |
| --- | --- | --- | --- | --- |
|  |  | x | y | z |
| ***Subcortical network*** |  |  |  |  |
| IC 34 (0.977)^2^ |  |  |  |  |
| R putamen | 21.85 | 18 | 13.5 | -4.5 |
| L putamen | 20.32 | -15 | 13.5 | -3 |
| IC 45 (0.973) |  |  |  |  |
| Thalamus | 20.18 | 13.5 | -19.5 | 9 |
| IC 50 (0.974) |  |  |  |  |
| R putamen | 21.91 | 25.5 | 4.5 | 3 |
| L putamen | 20.29 | -25.5 | 6 | 1.5 |
| ***Auditory network*** |  |  |  |  |
| IC 43 (0.975) |  |  |  |  |
| R superior temporal gyrus | 21.22 | 52.5 | -28.5 | 12 |
| L superior temporal gyrus | 19.72 | -51 | -21 | 10.5 |
| IC 61 (0.968) |  |  |  |  |
| R middle temporal gyrus | 18.83 | 51 | -37.5 | 3 |
| IC 86 (0.958) |  |  |  |  |
| L middle temporal gyrus | 22.39 | -54 | -40.5 | 6 |
| ***Visual network*** |  |  |  |  |
| IC 8 (0.982) |  |  |  |  |
| L middle occipital gyrus | 19.49 | -28.5 | -88.5 | 4.5 |
| IC 18 (0.979) |  |  |  |  |
| Middle occipital gyrus | 19.82 | -31.5 | -69 | 7.5 |
| IC 25 (0.978) |  |  |  |  |
| R calcarine gyrus | 22.23 | 7.5 | -78 | 3 |
| IC 30 (0.978) |  |  |  |  |
| L fusiform gyrus | 19.88 | -25.5 | -61.5 | -9 |
| R fusiform gyrus | 17.96 | 28.5 | -60 | -7.5 |
| IC 32 (0.977) |  |  |  |  |
| Middle occipital gyrus | 17.99 | 33 | -67.5 | 1.5 |
| IC 33 (0.976) |  |  |  |  |
| R cuneus | 19.98 | 6 | -79.5 | 21 |
| IC 57 (0.969) |  |  |  |  |
| R middle temporal gyrus | 16.21 | 52.5 | -58.5 | 6 |
| L middle temporal gyrus | 16.02 | -48 | -61.5 | 13.5 |
| IC 58 (0.967) |  |  |  |  |
| L calcarine gyrus | 22.99 | -12 | -61.5 | 6 |
| IC 79 (0.908) |  |  |  |  |
| L cuneus | 18.92 | -16.5 | -60 | 19.5 |
| R fusiform gyrus | 11.21 | 27 | -39 | -12 |
| ***Somatomotor network*** |  |  |  |  |
| IC 6 (0.983) |  |  |  |  |
| R supplementary motor area | 22.57 | 1.5 | -22.5 | 60 |
| IC 10 (0.983) |  |  |  |  |
| R postcentral gyrus | 24.87 | 55.5 | -7.5 | 27 |
| L postcentral gyrus | 24.17 | -52.5 | -9 | 33 |
| IC 31 (0.977) |  |  |  |  |
| R postcentral gyrus | 20.23 | 42 | -27 | 46.5 |
| IC 36 (0.975) |  |  |  |  |
| L postcentral gyrus | 21.28 | -34.5 | -22.5 | 51 |
| R precentral gyrus | 10.01 | 39 | -16.5 | 49.5 |
| IC 55 (0.971) |  |  |  |  |
| R supplementary motor area | 15.99 | 1.5 | -1.5 | 49.5 |
| IC 62 (0.971) |  |  |  |  |
| R postcentral gyrus | 14.95 | 24 | -42 | 58.5 |
| IC 75 (0.949) |  |  |  |  |
| L inferior parietal lobule | 18.17 | -52.5 | -27 | 37.5 |
| R inferior parietal lobule | 11.31 | 45 | -39 | 51 |
| IC 90 (0.914) |  |  |  |  |
| L supplementary motor area | 22.68 | -3 | 10.5 | 52.5 |
| ***Cognitive control network*** |  |  |  |  |
| IC 15 (0.981) |  |  |  |  |
| L superior frontal gyrus | 15.47 | -25.5 | 52.5 | 0 |
| IC 42 (0.975) |  |  |  |  |
| L angular gyrus | 18.57 | -45 | -61.5 | 45 |
| L inferior frontal gyrus | 14.25 | -42 | 46.5 | 1.5 |
| L superior medial frontal gyrus | 10.44 | -3 | 34.5 | 36 |
| IC 44 (0.976) |  |  |  |  |
| R inferior parietal lobule | 20.6 | 49.5 | -54 | 42 |
| R middle frontal gyrus | 12.61 | 30 | 19.5 | 55.5 |
| R middle cingulate cortex | 11.11 | 6 | -39 | 39 |
| IC 46 (0.972) |  |  |  |  |
| R angular gyrus | 18.75 | 30 | -61.5 | 42 |
| L middle occipital gyrus | 16.7 | -22.5 | -64.5 | 36 |
| IC 48 (0.979) |  |  |  |  |
| R middle frontal gyrus | 16.71 | 31.5 | 54 | 16.5 |
| L middle frontal gyrus | 15.05 | -28.5 | 49.5 | 10.5 |
| IC 67 (0.960) |  |  |  |  |
| R inferior frontal gyrus | 20.03 | 49.5 | 15 | 24 |
| L inferior frontal gyrus | 14.17 | -40.5 | 13.5 | 28.5 |
| IC 70 (0.948) |  |  |  |  |
| L inferior parietal lobule | 15.77 | -52.5 | -48 | 37.5 |
| R supramarginal gyrus | 15.74 | 49.5 | -45 | 24 |
| IC 81 (0.943) |  |  |  |  |
| R insula | 17.95 | 39 | 19.5 | -1.5 |
| L insula | 16.99 | -37.5 | 13.5 | -1.5 |
| IC 82 (0.931) |  |  |  |  |
| L superior medial frontal gyrus | 17.83 | -1.5 | 42 | 48 |
| IC 94 (0.771) |  |  |  |  |
| L hippocampus | 17.91 | -28.5 | -24 | -9 |
| Superior temporal gyrus | 11.48 | 48 | 3 | -13.5 |
| ***Default mode network*** |  |  |  |  |
| IC 24 (0.980) |  |  |  |  |
| R precuneus | 23.05 | 3 | -58.5 | 48 |
| IC 29 (0.976) |  |  |  |  |
| L precuneus | 25.45 | -4.5 | -69 | 33 |
| IC 54 (0.969) |  |  |  |  |
| L anterior cingulate cortex | 21.49 | 1.5 | 37.5 | 13.5 |
| IC 66 (0.960) |  |  |  |  |
| R precuneus | 26.48 | 1.5 | -54 | 25.5 |
| L angular gyrus | 16.18 | -49.5 | -66 | 30 |
| R angular gyrus | 14.26 | 45 | -60 | 28.5 |
| IC 99 (0.581) |  |  |  |  |
| L precuneus | 20.32 | -4.5 | -46.5 | 9 |
| ***Cerebellar network*** |  |  |  |  |
| IC 14 (0.979) |  |  |  |  |
| L cerebellum | 18.74 | -4.5 | -51 | -45 |
| IC 16 (0.983) |  |  |  |  |
| R cerebellum crus1 | 18.29 | 37.5 | -67.5 | -27 |
| IC 49 (0.979) |  |  |  |  |
| L cerebellum crus1 | 14.61 | -31.5 | -66 | -36 |

Abbreviations: RSN, resting-state network; T_max_, maximum cluster t-statistic; L, left; R, right.

^1^Coordinate (mm) of cluster peak activation in MNI space.

^2^Component number (Quality index - I_q_) indicated
