# Supplementary material for: Risperidone Effects on Brain Dynamic Connectivity—A Prospective Resting-State fMRI Study in Schizophrenia
Source: Front Psychiatry. 2017 Feb 6;8:14. doi: 10.3389/fpsyt.2017.00014 (PMC5292583; doi:10.3389/fpsyt.2017.00014)
Supplement: Supplementary file 2 [file Table_2.DOCX]

Table S2: Intraclass Correlation Coefficients for Controls Over Time (*n*=19)

|  | **ICC^1^** | **Cronbach’s Alpha** | ***F*-statistic** | ***p*-value** |
| --- | --- | --- | --- | --- |
| ***Mean Dwell Time*** |  |  |  |  |
| State 1 | 0.192 | 0.202 | 1.252 | 0.319 |
| State 2 | 0.714 | 0.703 | 3.367 | 0.007 |
| State 3 | -0.432 | -0.490 | 0.671 | 0.797 |
| ***Fraction of Time*** |  |  |  |  |
| State 1 | 0.485 | 0.496 | 1.984 | 0.078 |
| State 2 | 0.763 | 0.754 | 4.058 | 0.002 |
| State 3 | 0.022 | 0.023 | 1.024 | 0.480 |

Abbreviations: ICC, Intraclass correlation coefficient

^1^Average ICC values reported
