# Supplementary material for: Risperidone Effects on Brain Dynamic Connectivity—A Prospective Resting-State fMRI Study in Schizophrenia
Source: Front Psychiatry. 2017 Feb 6;8:14. doi: 10.3389/fpsyt.2017.00014 (PMC5292583; doi:10.3389/fpsyt.2017.00014)
Supplement: Supplementary file 3 [file Table_3.DOCX]

Table S3: Group Comparisons of State Statistics

|  | **Mean±SD** | | ***t*-statistic** | ***p*-value** |
| --- | --- | --- | --- | --- |
|  | Control | |  |  |
|  | Baseline (HC) | Week 6 (HC) |  |  |
| ***Mean Dwell Time*** |  |  |  |  |
| State 1 | 39.526±8.869 | 26.105±4.088 | 1.458 | 0.162 |
| State 2 | 10.118±3.329 | 9.803±2.952 | 0.1049 | 0.918 |
| State 3 | 11.740±1.457 | 16.298±2.079 | -1.641 | 0.118 |
| ***Fraction of Time*** |  |  |  |  |
| State 1 | 0.564±0.068 | 0.451±0.073 | 1.374 | 0.186 |
| State 2 | 0.164±0.058 | 0.176±0.060 | -0.243 | 0.811 |
| State 3 | 0.273±0.039 | 0.373±0.057 | -1.461 | 0.161 |
|  | Control vs. Schizophrenia | |  |  |
|  | Baseline (HC) | Baseline (SZ) |  |  |
| ***Mean Dwell Time*** |  |  |  |  |
| State 1 | 48.202±7.414 | 28.220±5.283 | 2.172 | 0.033* |
| State 2 | 8.093±2.074 | 10.543±3.163 | -0.655 | 0.515 |
| State 3 | 11.700±1.309 | 21.153±3.097 | -2.869 | 0.006* |
| ***Fraction of Time*** |  |  |  |  |
| State 1 | 0.611±0.051 | 0.418±0.053 | 2.635 | 0.010* |
| State 2 | 0.116±0.034 | 0.151±0.035 | -0.719 | 0.475 |
| State 3 | 0.273±0.035 | 0.431±0.037 | -3.094 | 0.003* |
|  | Control vs. Schizophrenia | |  |  |
|  | Baseline (HC) | Week 1 (SZ) |  |  |
| ***Mean Dwell Time*** |  |  |  |  |
| State 1 | 48.202±7.414 | 40.701±7.722 | 0.697 | 0.488 |
| State 2 | 8.093±2.074 | 11.218±2.457 | -0.979 | 0.331 |
| State 3 | 11.700±1.309 | 19.667±2.038 | -3.396 | 0.001* |
| ***Fraction of Time*** |  |  |  |  |
| State 1 | 0.611±0.051 | 0.448±0.058 | 2.130 | 0.037* |
| State 2 | 0.116±0.034 | 0.144±0.030 | -0.593 | 0.555 |
| State 3 | 0.273±0.035 | 0.409±0.043 | -2.480 | 0.016* |
|  | Control vs. Schizophrenia | |  |  |
|  | Baseline (HC) | Week 6 (SZ) |  |  |
| ***Mean Dwell Time*** |  |  |  |  |
| State 1 | 48.202±7.414 | 44.236±7.971 | 0.356 | 0.723 |
| State 2 | 8.093±2.074 | 5.507±1.433 | 0.932 | 0.355 |
| State 3 | 11.700±1.309 | 17.299±2.542 | -2.130 | 0.037* |
| ***Fraction of Time*** |  |  |  |  |
| State 1 | 0.611±0.051 | 0.539±0.072 | 0.851 | 0.399 |
| State 2 | 0.116±0.034 | 0.093±0.028 | 0.480 | 0.633 |
| State 3 | 0.273±0.035 | 0.368±0.054 | -1.548 | 0.127 |
|  | Schizophrenia | |  |  |
|  | Baseline (SZ) | Week 1 (SZ) |  |  |
| ***Mean Dwell Time*** |  |  |  |  |
| State 1 | 27.112±5.525 | 40.701±7.722 | -1.639 | 0.112 |
| State 2 | 10.813±3.580 | 11.218±2.457 | -0.089 | 0.930 |
| State 3 | 20.976±3.507 | 19.667±2.038 | 0.341 | 0.736 |
| ***Fraction of Time*** |  |  |  |  |
| State 1 | 0.421±0.057 | 0.447±0.058 | -0.384 | 0.704 |
| State 2 | 0.149±0.038 | 0.144±0.030 | 0.116 | 0.909 |
| State 3 | 0.430±0.041 | 0.409±0.043 | 0.394 | 0.696 |
|  | Schizophrenia | |  |  |
|  | Baseline (SZ) | Week 6 (SZ) |  |  |
| ***Mean Dwell Time*** |  |  |  |  |
| State 1 | 23.438±5.241 | 43.507±8.291 | -2.757 | 0.012* |
| State 2 | 11.482±4.461 | 5.746±1.475 | 1.193 | 0.246 |
| State 3 | 23.520±4.231 | 17.791±2.605 | 1.412 | 0.172 |
| ***Fraction of Time*** |  |  |  |  |
| State 1 | 0.398±0.062 | 0.521±0.073 | -1.745 | 0.095 |
| State 2 | 0.150±0.046 | 0.098±0.029 | 0.894 | 0.381 |
| State 3 | 0.452±0.045 | 0.382±0.054 | 1.368 | 0.185 |
|  | Schizophrenia | |  |  |
|  | Week 1 (SZ) | Week 6 (SZ) |  |  |
| ***Mean Dwell Time*** |  |  |  |  |
| State 1 | 43.394±9.190 | 42.644±8.630 | 0.068 | 0.946 |
| State 2 | 11.220±2.973 | 6.008±1.519 | 1.679 | 0.108 |
| State 3 | 20.228±2.561 | 18.463±2.634 | 0.540 | 0.595 |
| ***Fraction of Time*** |  |  |  |  |
| State 1 | 0.457±0.067 | 0.500±0.073 | -0.632 | 0.534 |
| State 2 | 0.135±0.034 | 0.102±0.030 | 0.887 | 0.385 |
| State 3 | 0.408±0.052 | 0.398±0.054 | 0.170 | 0.867 |

Abbreviations: SD, standard deviation; HC, healthy control; SZ, schizophrenia
